# Protein arginine deiminase 2 (PAD2) modulates the polarization of THP-1 macrophages to the anti-inflammatory M2 phenotype

**DOI:** 10.1186/s12950-022-00317-8

**Published:** 2022-11-18

**Authors:** Aneta Stachowicz, Rakhi Pandey, Niveda Sundararaman, Vidya Venkatraman, Jennifer E. Van Eyk, Justyna Fert-Bober

**Affiliations:** 1 Chair of Pharmacology, Jagiellonian University Medical College, Krakow, Poland; 2 Advanced Clinical Biosystems Research Institute, Smidt Heart Institute, Cedars-Sinai Medical Center, CA Los Angeles, USA; 3 Advanced Clinical Biosystems Research Institute, Precision Biomarker Laboratories, Cedars-Sinai Medical Center, Los Angeles, CA USA

**Keywords:** Citrullination, Protein arginine deiminases, Proteomics, Macrophage polarization, Posttranslational modifications

## Abstract

**Background:**

Macrophages are effector cells of the innate immune system that undergo phenotypical changes in response to organ injury and repair. These cells are most often classified as proinflammatory M1 and anti-inflammatory M2 macrophages. Protein arginine deiminase (PAD), which catalyses the irreversible conversion of protein-bound arginine into citrulline, is expressed in macrophages. However, the substrates of PAD and its role in immune cells remain unclear. This study aimed to investigate the role of PAD in THP-1 macrophage polarization to the M1 and M2 phenotypes and identify the citrullinated proteins and modified arginines that are associated with this biological switch using mass spectrometry.

**Results:**

Our study showed that PAD2 and, to a lesser extent, PAD1 and PAD4 were predominantly expressed in M1 macrophages. We showed that inhibiting PAD expression with BB-Cl-amidine decreased macrophage polarization to the M1 phenotype (TNF-α, IL-6) and increased macrophage polarization to the M2 phenotype (MRC1, ALOX15). This process was mediated by the downregulation of proteins involved in the NF-κβ pathway. Silencing PAD2 confirmed the activation of M2 macrophages by increasing the antiviral innate immune response and interferon signalling. A total of 192 novel citrullination sites associated with inflammation, cell death and DNA/RNA processing pathways were identified in M1 and M2 macrophages.

**Conclusions:**

We showed that inhibiting PAD activity using a pharmacological inhibitor or silencing PAD2 with PAD2 siRNA shifted the activation of macrophages towards the M2 phenotype, which can be crucial for designing novel macrophage-mediated therapeutic strategies. We revealed a major citrullinated proteome and its rearrangement following macrophage polarization, which after further validation could lead to significant clinical benefits for the treatment of inflammation and autoimmune diseases.

**Supplementary Information:**

The online version contains supplementary material available at 10.1186/s12950-022-00317-8.

## Background

The protein arginine deiminase (PAD) family of enzymes catalyses the conversion of positively charged arginine residues within a protein to neutrally charged citrulline in a hydrolytic reaction termed citrullination or deamination [1, 2]. This irreversible reaction has attracted attention due to its involvement in various physiologic and pathologic conditions [3, 4]. On the global level PAD isoforms have been reported to be widespread in inflammatory cells [5, 6]. It has been shown that the activation of PAD4 in neutrophils triggers histone citrullination and chromatin decondensation, resulting in neutrophil extracellular trap (NET) formation during host defence, inflammation, and autoimmunity [7]. Similarly, PAD2 has been shown to mediate macrophage NETosis (ETosis or METosis) [8] and pyroptosis, an inflammatory form of macrophage death [9]. Notably, it has been shown that PAD2 may be involved in THP-1 monocyte differentiation to macrophages [10]; however, neither the role of PAD in macrophage polarization to proinflammatory and anti-inflammatory phenotypes nor the proteins that are specifically citrullinated have been investigated.

Macrophages play a pivotal role in innate immunity and are involved in the development of low-grade chronic inflammation in many disorders, such as atherosclerosis [11], rheumatoid arthritis [12] and Alzheimer’s disease [13]. Tissue-resident macrophages are very plastic and can be classified according to two main phenotypes: proinflammatory M1 macrophages (classically activated) and anti-inflammatory M2 macrophages (alternatively activated). M1 macrophages emerge in response to lipopolysaccharide (LPS) and interferon gamma (IFN-γ), while M2 macrophages are generated in response to interleukin-4 (IL-4) and interleukin-13 (IL-13) [14]. In general, M1 macrophages are characterized by the production of nitric oxide (NO) and inflammatory cytokines (i.e., interleukin-1 beta (IL-1β,) tumour necrosis factor α (TNF-α), and interleukin-6 (IL-6)) and are responsible for the clearance of pathogens, whereas M2 macrophages release anti-inflammatory cytokines and play a role in the resolution of inflammation, tissue repair and wound healing [15, 16]. Notably, macrophage class switching from proinflammatory M1 to anti-inflammatory M2 cells could be a potential therapeutic target in the treatment of chronic inflammatory diseases [17].

Thus, we aimed to investigate the role of PAD during THP-1 macrophage polarization to proinflammatory M1 and anti-inflammatory M2 phenotypes. Furthermore, we aimed to determine the citrullinated proteins and modified arginines that are associated with this biological switch using mass spectrometry. Revealing the major citrullinated proteome and its rearrangement following macrophage polarization could lead to significant clinical benefits for the treatment of inflammation and autoimmune diseases.

## Results

### PAD2 is the most abundant PAD isoform in THP-1 macrophages

**Fig. 1:**
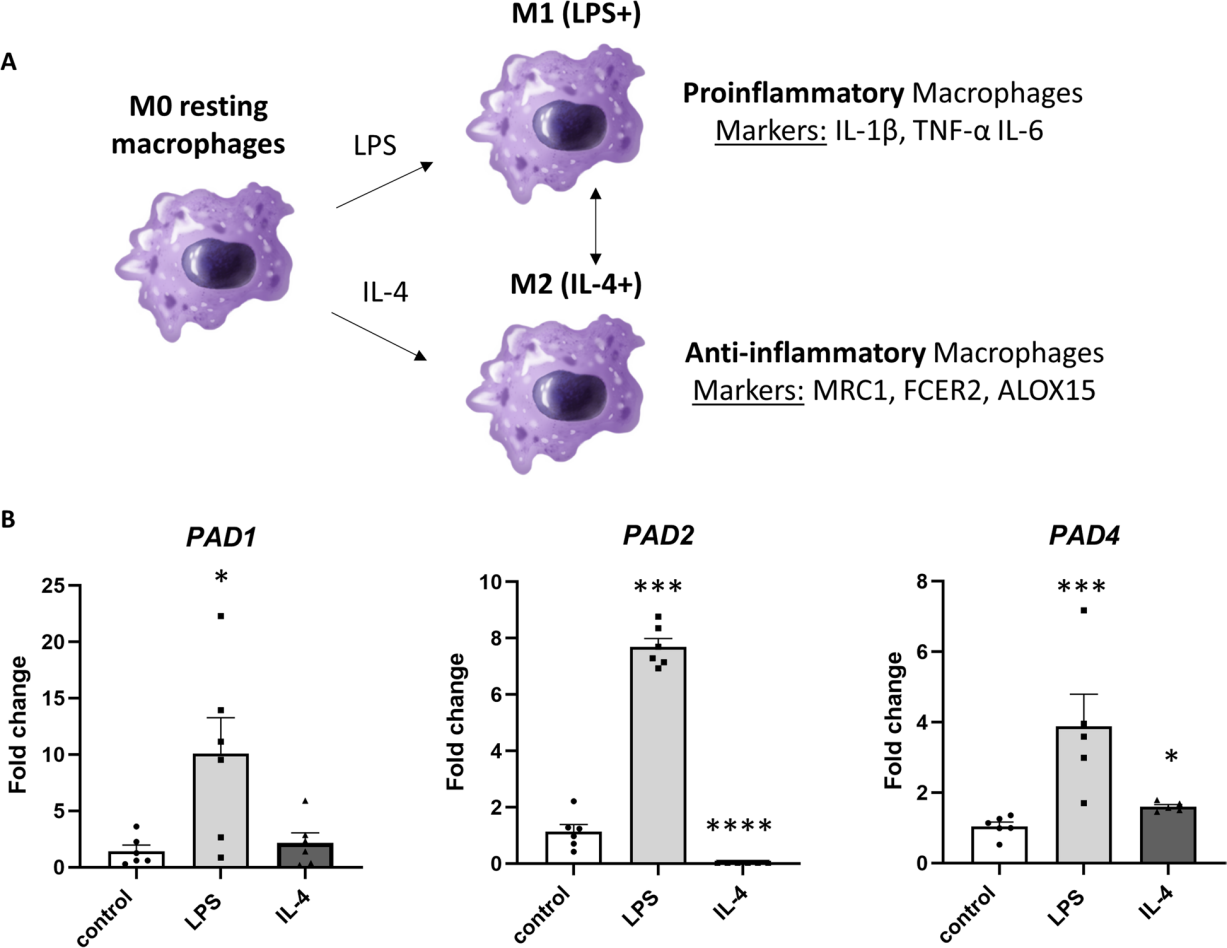
Protein arginine deiminase (PAD) expression in macrophages polarized to proinflammatory (M1) and anti-inflammatory (M2) phenotypes. PAD1, PAD2 and PAD4 expression was upregulated in M1 macrophages, while PAD2 was strongly downregulated in M2 macrophages. mRNA expression of PAD1, PAD2 and PAD4 in THP-1 macrophages treated with LPS (M1 phenotype) or IL-4 (M2 phenotype) for 48 h. mRNA expression of PAD3 was not present in THP-1 macrophages. Mean ± SEM; *p < 0.05, ***p < 0.001, ****p < 0.0001 compared to control; *n* = 5

In this study, differently polarized human THP-1 macrophages were analyzed by quantitative proteomics to evaluate PAD role during macrophage polarization. We first investigated the polarization of THP-1 macrophages to proinflammatory and anti-inflammatory phenotypes as presented in Fig. 1A. We then focused on the PAD1, PAD2, PAD3 and PAD4 mRNA expression levels that were measured by quantitative RT-PCR. The most abundant PAD isoforms in resting THP-1 macrophages (control) were PAD2 (cycle threshold (Ct) = 25.87 ± 0.16) and PAD1 (Ct = 31.06 ± 0.33), followed by PAD4 (Ct = 35.47 ± 0.21). We did not detect any mRNA expression of PAD3. PAD expression was altered in proinflammatory and anti-inflammatory THP-1 macrophages. We observed statistically significant increases in PAD1 (fold change (FC) = 10.06 ± 3.19 vs. FC = 1.43 ± 0.52 in control, *p* < 0.05), PAD2 (FC = 7.7 ± 0.3 vs. FC = 1.14 ± 0.25 in control, *p* < 0.001), and PAD4 (FC = 3.34 ± 0.92 vs. FC = 1.04 ± 0.12 in control, *p* < 0.001) mRNA expression levels in LPS-treated macrophages (proinflammatory M1 type). Interestingly, the mRNA expression of PAD2 (FC = 0.0299 ± 0.0007 vs. FC = 1.14 ± 0.25 in control, *p* < 0.0001) was highly downregulated in IL-4-treated THP-1 macrophages (anti-inflammatory M2 type), while the mRNA expression of PAD4 (FC = 1.47 ± 0.14 vs. FC = 1.04 ± 0.12 in control, *p* < 0.05) was upregulated (Fig. 1B).

### The pan-PAD inhibitor – BB-Cl-amidine decreases the polarization of THP-1 macrophages to the M1 phenotype and increases polarization to the M2 phenotype

**Fig. 2:**
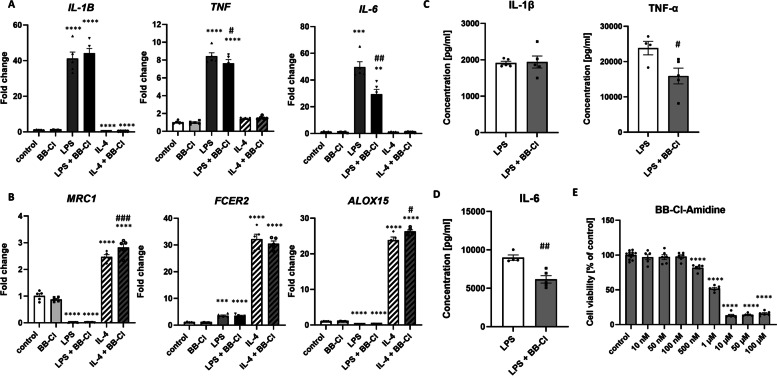
Influence of the pan-PAD inhibitor BB-Cl-amidine (BB-Cl) on the polarization of macrophages to the M1 and M2 phenotypes. BB-Cl decreased the polarization of THP-1 macrophages to the M1 phenotype and increased activation to the M2 phenotype. **A** mRNA expression of M1 markers (IL-1β, TNF-α, IL-6) in THP-1 macrophages treated with LPS (M1 phenotype) or IL-4 (M2 phenotype) for 48 h in the presence of BB-Cl. **B** mRNA expression of M2 markers (MRC1, FCER2, ALOX15) in THP-1 macrophages treated with LPS (M1 phenotype) or IL-4 (M2 phenotype) for 48 h in the presence of BB-Cl. **C** and **D** Concentration of proinflammatory cytokines (IL-1β, TNF-α, IL-6) in the cell supernatant of THP-1 macrophages treated with LPS for 48 h in the presence of BB-Cl. **E** The toxicity of BB-Cl-amidine in THP-1 macrophages was measured by MTT assays at 48 h. Mean ± SEM; **p < 0.01, ***p < 0.001, ****p < 0.0001 compared to control; #*p* < 0.05, ##*p* < 0.01, ###*p* < 0.001 compared to LPS or IL-4; *n* = 5

The mRNA expression of PAD1, PAD2 and PAD4 differed between proinflammatory and anti-inflammatory THP-1 macrophages and was particularly upregulated in proinflammatory M1 cells. To determinate whether PAD activation affects the switch in THP-1 macrophages to the M1 and M2 phenotypes, we used BB-Cl-amidine, which irreversibly inhibits the activity of all PADs [18]. In our experiments, we chose a dose of BB-Cl-amidine equal to 100 nM based on the results of the MTT cell viability assay (Fig. 2 E).

BB-Cl-amidine treatment of THP-1 macrophages significantly reduced the mRNA expression of proinflammatory markers, such as TNF-α (FC = 7.64 ± 0.4 vs. FC = 8.43 ± 0.4 in LPS-treated cells, *p* < 0.05) and IL-6 (FC = 29.41 ± 3.61 vs. FC = 49.75 ± 3.95 in LPS-treated cells, *p* < 0.01) in M1 cells (Fig. 2A) and upregulated the mRNA expression of anti-inflammatory markers, including MRC1 (FC = 2.82 ± 0.13 vs. FC = 2.47 ± 0.08 in IL-4-treated cells, *p* < 0.001) and ALOX15 (FC = 26.31 ± 0.43 vs. FC = 23.86 ± 0.87 in IL-4-treated cells, *p* < 0.05), in M2 cells (Fig. 2B). Furthermore, the levels of the proinflammatory cytokines TNF-α (15,928 ± 2240 vs. 23,835 ± 1912 in LPS-treated cells, *p* < 0.05) and IL-6 (6164 ± 483 vs. 8993 ± 346 in LPS-treated cells, *p* < 0.01) were decreased in the cell supernatant of M1 macrophages after treatment with BB-Cl-amidine (Fig. 2C and D).

### Inhibition of PAD activity alters proinflammatory pathways in THP-1 macrophages

In vitro experiments showed that inhibiting PAD activity in THP-1 macrophages (BB-Cl-amidine treatment) decreased the switch in macrophages to the M1 type and augmented polarization to the M2 type. To further understand the mechanism involved and identify relevant citrullinated proteins, we analysed the proteomic profile of M1 and M2 cell lysates by MS.

Liquid chromatography – tandem mass spectrometry (LC-MS/MS) measurements operated in DDA mode identified 22,211 peptides, providing a macrophages spectral library composed of 4832 protein groups. The obtained library was used to analyse DIA runs in Spectronaut. Spectral library recovery was 85%, and the median protein group CV was approximately 20% (Supplemental Fig. 1), which allowed for the calculation of a significant cut-off equal to a fold change of 1.35 (statistical power greater than 80%). A summary of the quality control for the LC-MS/MS runs is shown in Supplemental Fig. 1.

**Fig. 3:**
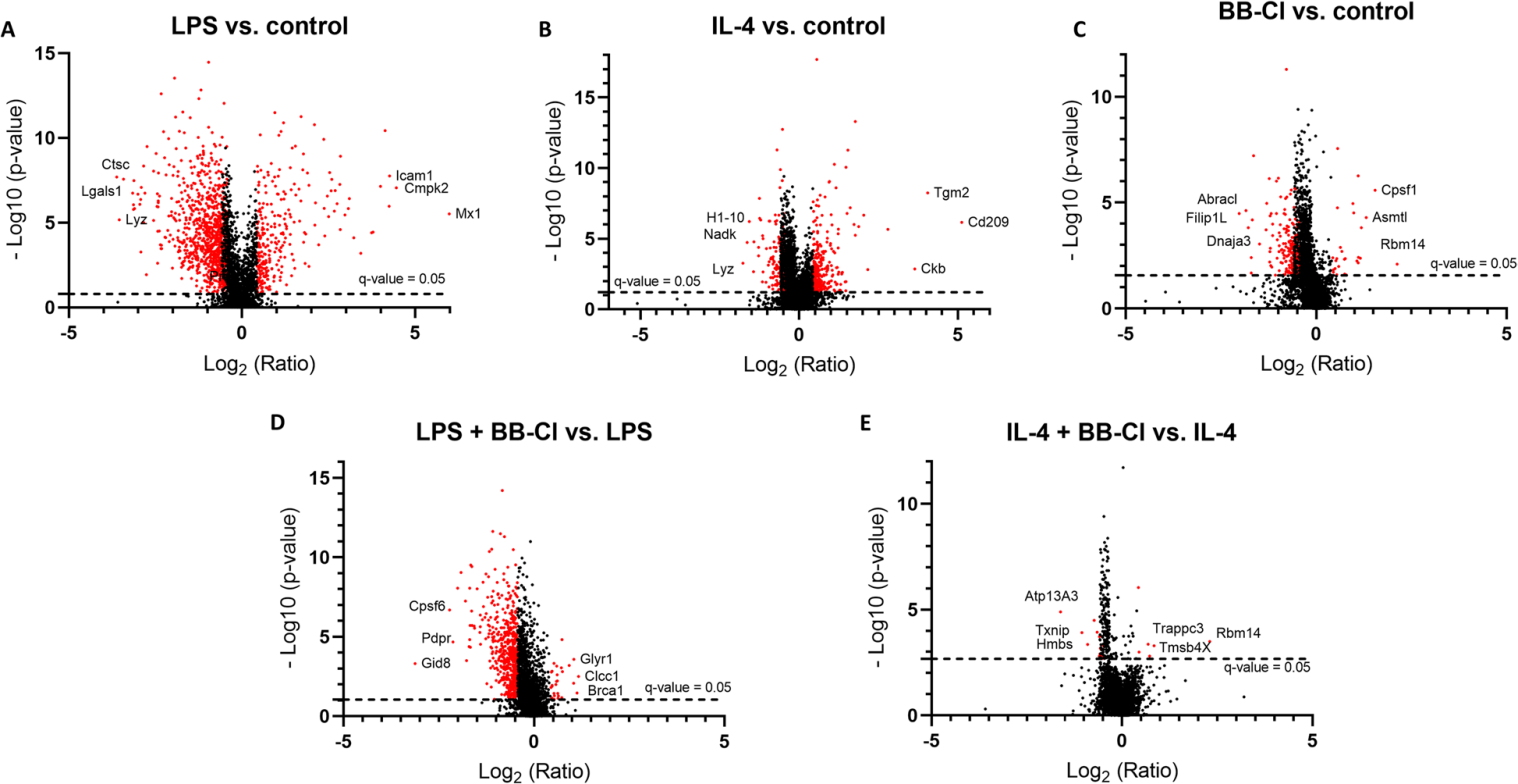
Proteomics results of THP-1 macrophages activated to the M1 and M2 phenotypes in the presence of the pan-PAD inhibitor BB-Cl-amidine. The acquired data allowed for the identification and quantitation of 3005 protein groups across all biological conditions. Volcano plot of differentially expressed proteins showing the log2 ratio of protein expression versus -log10 *p* value. **A** LPS-treated macrophages compared to control, **B** IL-4-treated macrophages compared to control, **C** BB-Cl-treated macrophages compared to control, and **D** LPS-treated macrophages stimulated with BB-Cl compared to LPS, D) IL-4-treated macrophages stimulated with BB-Cl compared to IL-4. The most upregulated and downregulated proteins are shown. q value< 0.05; *n* = 5

**Fig. 4:**
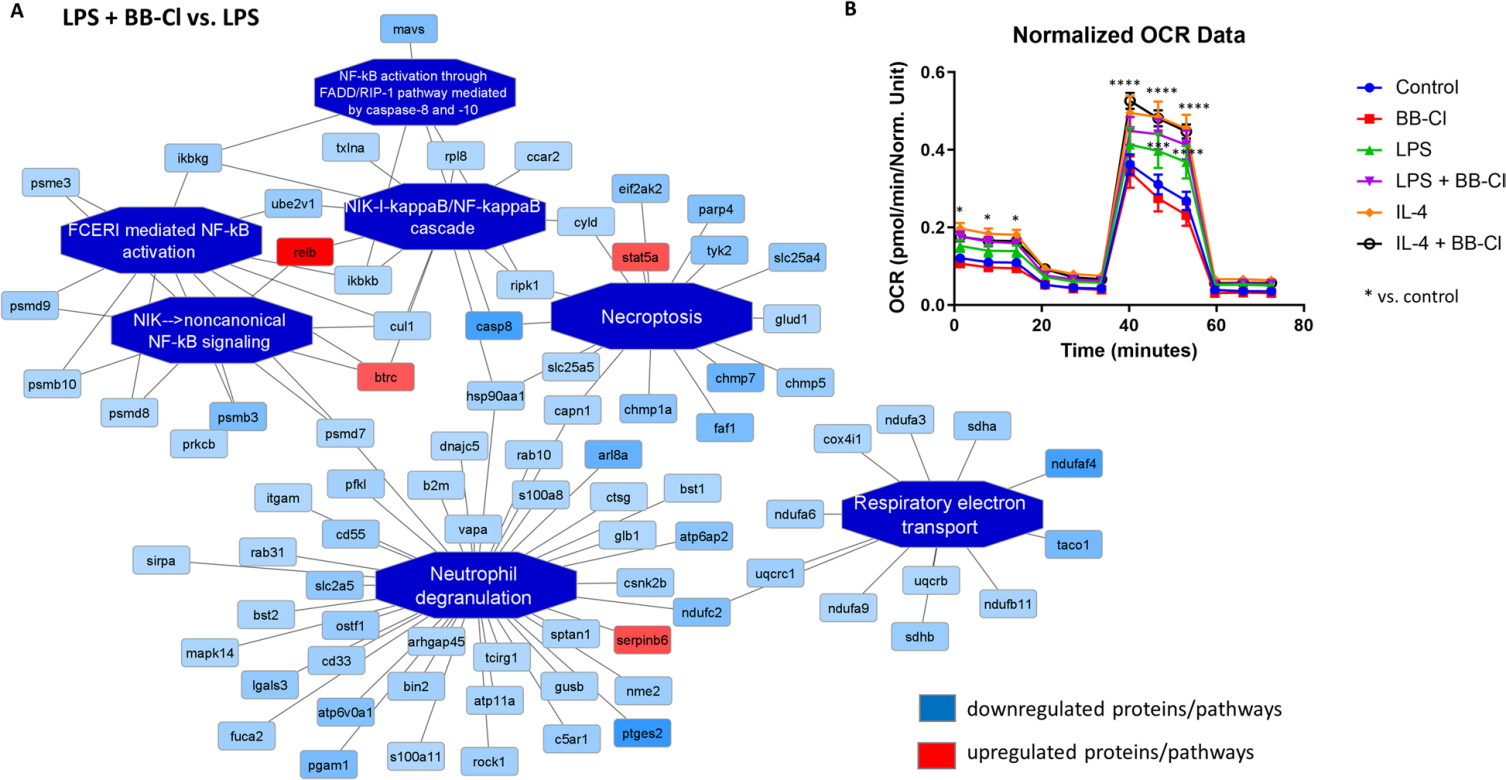
Bioinformatic functional network analysis of THP-1 macrophages treated with the pan-PAD inhibitor BB-Cl-amidine. Inhibiting PAD in proinflammatory macrophages was associated with the downregulation of proteins involved in the activation of NF-κβ signalling. **A** Enriched functional network generated by PINE (Protein Interaction Network Extractor) in THP-1 macrophages treated with LPS and BB-Cl compared to LPS-treated cells (M1 proinflammatory macrophages). Activated pathways are shown as orange central nodes and inhibited pathways are shown as blue central nodes, along with red (upregulated) or blue (downregulated) protein nodes. **B** The oxygen consumption rate (OCR) was normalized to the protein level and measured by the Seahorse XF96 Mito Stress Test in THP-1 macrophages treated with LPS (M1 phenotype) or IL-4 (M2 phenotype) for 24 h in the presence of BB-Cl. Mean ± SEM; *p < 0.05, ***p < 0.001, ****p < 0.0001 compared to control; *n* = 5

The acquired data allowed for the identification and quantitation of 3005 protein groups across all biological conditions. A total of 254 proteins were altered in BB-Cl-amidine–treated THP-1 macrophages compared to the control; 1160 proteins were altered in LPS-stimulated cells compared to control as well as 435 proteins were differentially expressed in IL-4 stimulated macrophages in comparison to the control (Fig. 3). BB-Cl-amidine administration to proinflammatory M1 macrophages (LPS stimulated) led to 554 differentially expressed proteins compared to LPS-treated cells, while only 24 proteins were altered in anti-inflammatory M2 macrophages (IL-4 stimulated) treated with BB-Cl-amidine in comparison to IL-4-treated macrophages (Fig. 3). Using cut-offs of q < 0.05 and log_2_FC ≤ 0.433, we found decreased levels of several proteins, including NF-kappa-B essential modulator (IKBKG), protein kinase C beta type (PRKCB), inhibitor of nuclear factor kappa-B kinase subunit beta (IKK2), ubiquitin-conjugating enzyme E2 variant 1 (UBE2V1), interferon-induced 35 kDa protein (IFI35), mitochondrial antiviral-signalling protein (MAVS), double-stranded RNA-activated protein kinase (EIF2AK2) and receptor-interacting serine/threonine-protein kinase 1 (RIPK1) (Fig. 4A). Furthermore, we observed decreased expression of stimulator of interferon genes protein (STING1) in proinflammatory M1 and anti-inflammatory M2 macrophages after treatment with BB-Cl-amidine (FC = -1.92 and − 1.42, respectively) (Supplemental Table 1). In M2 macrophages, the administration of BB-Cl-amidine did not result in statistically significant changes in protein expression, except thioredoxin-interacting protein (TXNIP) (FC = -2.07) and caspase-1 (FC = -1.51), which were downregulated in M2 macrophages after BB-Cl administration (Supplemental Table 1).

Moreover, the expression levels of proteins that participate in oxidative phosphorylation - respiratory electron transport in mitochondria were diminished in BB-Cl-amidine-treated and LPS-stimulated macrophages (Fig. 4A). Interestingly, this effect was not reflected in the functional analysis of mitochondria, as we did not detect differences in the OCR in M1 and M2 macrophages treated with BB-Cl-amidine (Fig. 4B). The detailed list of differentially expressed proteins and their fold changes across all biological conditions are presented in Supplemental Table 1. Additionally, enriched pathway analyses performed by PINE on M1 (LPS+) and M2 (IL-4+) macrophages are depicted in Supplemental Fig. 2.

### Silencing PAD2 in THP-1 macrophages increases the activation of macrophages to the M2 phenotype

**Fig. 5:**
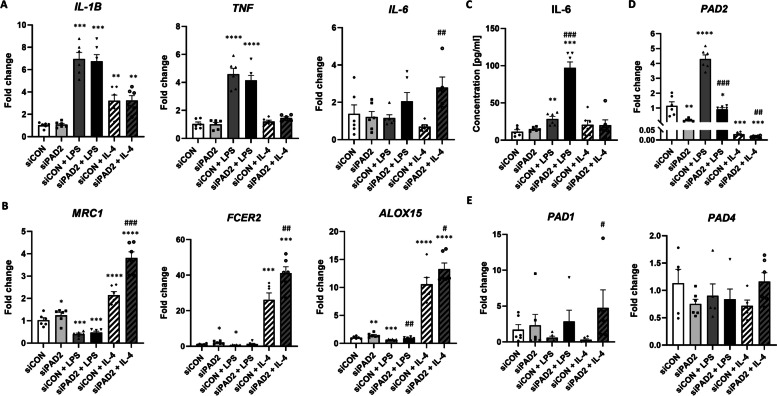
Influence of PAD2 silencing on the polarization of macrophages to the M1 and M2 phenotypes. Silencing PAD2 in THP-1 macrophages increased the activation of macrophages to the M2 phenotype. **A** mRNA expression of M1 markers (IL-1β, TNF-α, IL-6) in THP-1 macrophages treated with siPAD2 or scrambled siRNA control (siCON) and LPS (M1 phenotype) or IL-4 (M2 phenotype) for 48 h. **B** mRNA expression of M2 markers (MRC1, FCER2, ALOX15) in THP-1 macrophages treated with siPAD2 or scrambled siRNA control (siCON) and LPS (M1 phenotype) or IL-4 (M2 phenotype) for 48 h. **C** Concentrations of proinflammatory cytokines: IL-6 in the cell supernatant of THP-1 macrophages treated with siPAD2 or scrambled siRNA control (siCON) and LPS (M1 phenotype) or IL-4 (M2 phenotype) for 48 h. **D** mRNA expression of PAD2, PAD1 and PAD4 in THP-1 macrophages treated with siPAD2 or scrambled siRNA control (siCON) and LPS (M1 phenotype) or IL-4 (M2 phenotype) for 48 h. Mean ± SEM; *p < 0.05, **p < 0.01, ***p < 0.001, ****p < 0.0001 compared to control; #*p* < 0.05, ##*p* < 0.01, ###*p* < 0.001 compared to siCON + LPS or siCON + IL-4; *n* = 6

To further understand the mechanism by which PAD modulates macrophages polarization, we silenced PAD2 in macrophages, since PAD2 was the most highly expressed PAD isozyme in THP-1 macrophages. We confirmed the silencing of PAD2 (siPAD2) in control, LPS-treated and IL-4-treated macrophages by real-time PCR (Fig. 5D). PAD2 silencing did not influence the mRNA expression of PAD4, but PAD1 mRNA expression tended to be increased (FC = 2.42 ± 1.36 vs. FC = 0.61 ± 0.19 in siCON + LPS-treated cells, *p* > 0.05; FC = 3.98 ± 2.19 vs. FC = 0.31 ± 0.13 in siCON + IL-4-treated cells, *p* < 0.05) in comparison to cells treated with the scrambled siRNA control (siCON) and stimulated with either LPS or IL-4 (Fig. 5E). This result might reflect the compensatory role of PAD1 in THP-1 macrophages with silenced PAD2.

Silencing PAD2 in LPS-stimulated proinflammatory macrophages did not change the mRNA expression of the proinflammatory markers IL-1β and TNF-α (Fig. 5A). However, it increased the mRNA expression of IL-6 (FC = 2.79 ± 0.51 vs. FC = 0.7 ± 0.1 in siCON + IL-4-treated cells, *p* < 0.05) in M2 macrophages and tended to upregulate IL-6 expression in M1 macrophages (FC = 2.06 ± 0.46 vs. FC = 1.16 ± 0.17 in siCON + LPS-treated cells, *p* > 0.05) (Fig. 5A). This trend was also confirmed by ELISA analysis of the supernatant of LPS-stimulated THP-1 macrophages treated with siPAD2 (97.01 ± 8.03 vs. 28.56 ± 3.28 in siCON + LPS-treated cells, *p* < 0.001)(Fig. 5C). Interestingly, silencing PAD2 upregulated the mRNA expression of anti-inflammatory markers of M2 macrophages, including MRC1 (FC = 3.81 ± 0.28 vs. FC = 2.15 ± 0.16 in siCON + IL-4-treated cells, *p* < 0.001; FC = 1.24 ± 0.13 vs. FC = 1.03 ± 0.11 in siCON cells, *p* < 0.05), FCER2 (FC = 41.12 ± 3.60 vs. FC = 26.19 ± 3.86 in siCON + IL-4-treated cells, *p* < 0.01; FC = 2.08 ± 0.32 vs. FC = 1.08 ± 0.18 in siCON cells, *p* < 0.05), and ALOX15 (FC = 13.29 ± 1.08 vs. FC = 10.57 ± 1.24 in siCON + IL-4-treated cells, *p* < 0.05; FC = 1.43 ± 0.16 vs. FC = 1.02 ± 0.09 in siCON cells, *p* < 0.01) in both control cells and IL-4 stimulated macrophages (Fig. 5B).

### Silencing PAD2 in THP-1 macrophages upregulates pathways related to interferon signalling

**Fig. 6:**
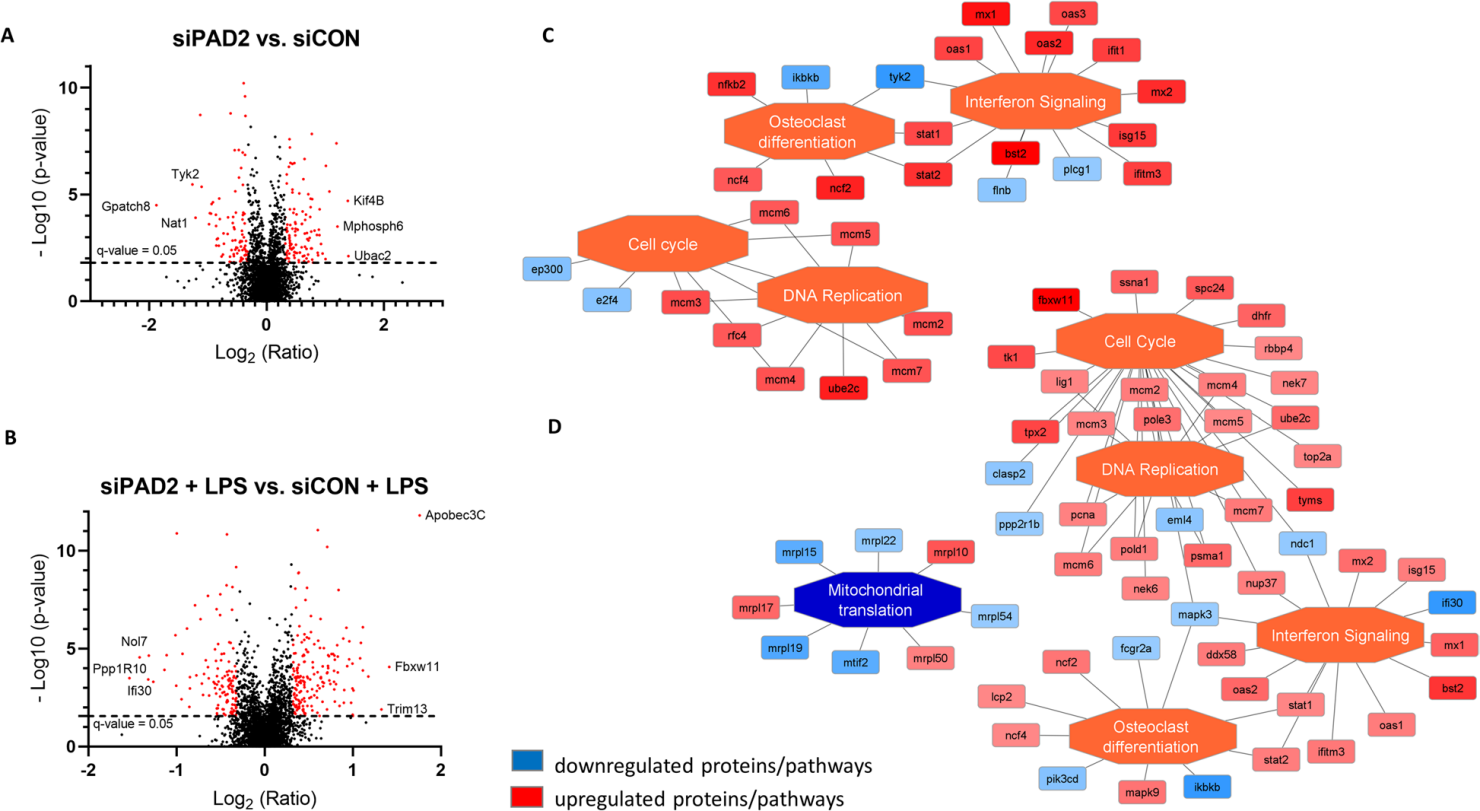
Proteomics results and functional network analysis of THP-1 macrophages with silenced PAD2 that were activated to the M1 phenotype. Silencing PAD2 upregulated pathways related to the antiviral innate immune response and interferon signalling. Volcano plot of differentially expressed proteins showing the log2 ratio of protein expression versus -log10 *p* value. **A** PAD2-silenced macrophages (siPAD2) compared to scrambled siRNA control (siCON) and **B** PAD2-silenced macrophages treated with LPS (siPAD2 + LPS) compared to scrambled siRNA control treated with LPS (siCON + LPS). The most upregulated and downregulated proteins are shown. **C** Enriched functional network in PAD2-silenced macrophages (siPAD2) compared to scrambled siRNA control (siCON), as generated by PINE. **D** Enriched functional network in PAD2 silenced macrophages (siPAD2 + LPS) treated with LPS compared to scrambled siRNA control treated with LPS (siCON + LPS), as generated by PINE. Activated pathways are shown as orange central nodes and inhibited pathways are shown as blue central nodes along with red (upregulated) or blue (downregulated) protein nodes. q value< 0.05; *n* = 6

MS analysis after silencing PAD2 in THP-1 macrophages identified 3820 protein groups across all biological conditions. Using cut-offs of q < 0.05 and log_2_FC ≤ 0.322, we found 205 proteins that were altered in THP-1 macrophages with silenced PAD2 compared to scrambled siRNA control cells and 306 proteins that were differentially expressed in LPS-stimulated, PAD2-silenced cells in comparison to LPS-stimulated, scrambled siRNA-treated control macrophages (Fig. 6A and B). The detailed list of differentially expressed proteins and their fold changes across all biological conditions are presented in Supplemental Table 2. The summary of quality control for LC-MS/MS runs is presented in Supplemental Fig. 3. Enriched pathway analysis showed the upregulation of interferon signalling, DNA replication and the cell cycle in PAD2-silenced THP-1 macrophages (Fig. 6C and D). Furthermore, proteins responsible for the antiviral innate immune response and interferon signalling, such as the interferon-induced GTP-binding proteins Mx1 and Mx2 (MX1 and MX2); 2′-5′-oligoadenylate synthases 1, 2 and 3 (OAS1, OAS2 and OAS3); interferon-induced transmembrane protein 3 (IFITM3); ubiquitin-like protein ISG15 (ISG15); bone marrow stromal antigen 2 (BST2) and the antiviral innate immune response receptor RIG-I (DDX58 or RIG-I), were increased in PAD2-silenced cells. Furthermore, our study revealed that in PAD2-silenced macrophages, the expression of HLA class II histocompatibility antigen gamma chain (CD74) was upregulated (FC = 2.1) in comparison to scrambled siRNA control cells (Supplemental Table 2).

### Protein citrullination landscape of THP-1 macrophages

**Fig. 7:**
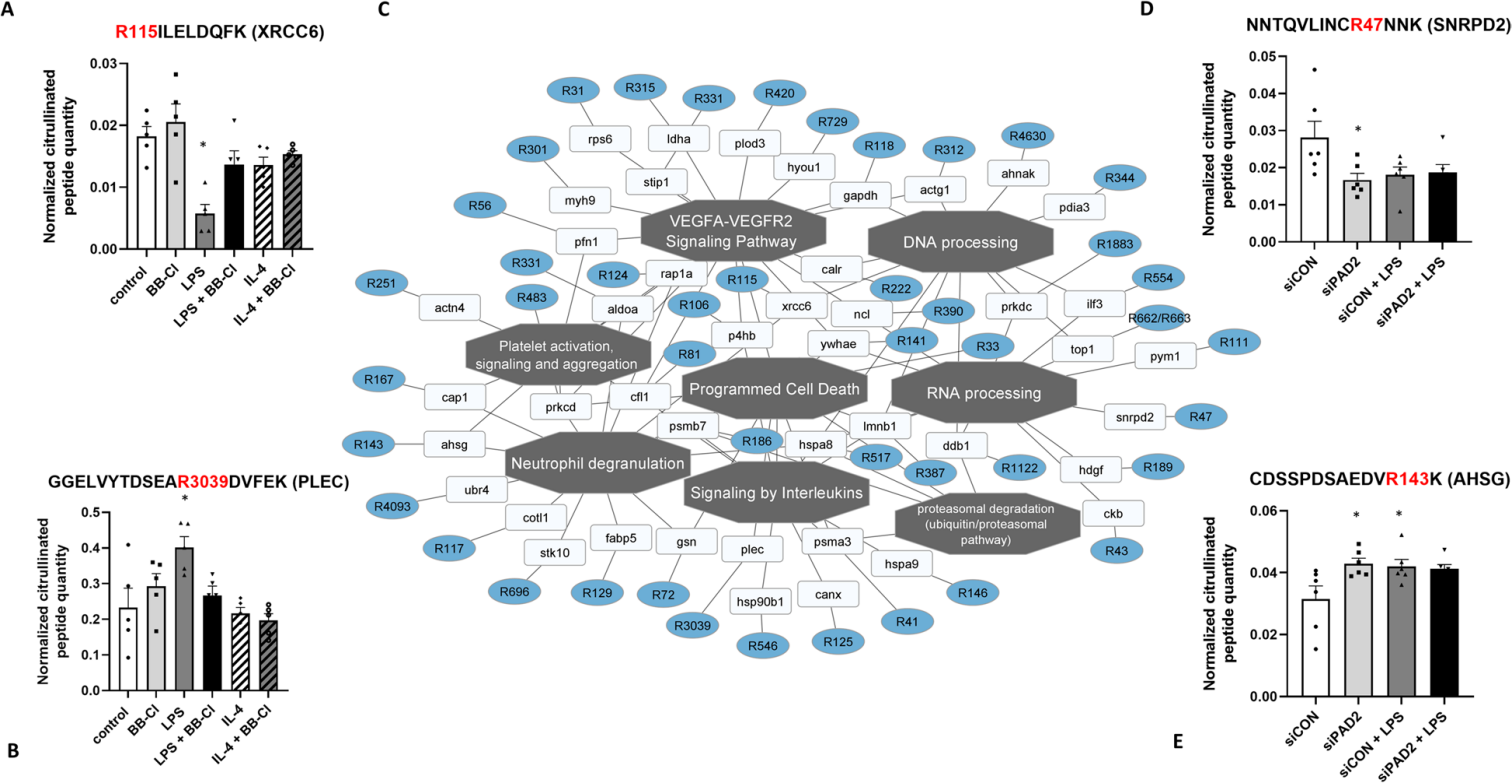
Citrullination landscape of THP-1 macrophages polarized to proinflammatory (M1) and anti-inflammatory phenotypes (M2). The identified and quantified citrullinated proteins were engaged in signalling by interleukins, neutrophil degranulation, programmed cell death, DNA and RNA processing, proteasomal degradation, platelet activation and VEGF signalling. **A** Normalized citrullinated peptide quantities of R115ILELDQFK (XRCC6) and **B** GGELVYTDSEAR3039DVFEK (PLEC) in THP-1 macrophages treated with LPS (M1 phenotype) or IL-4 (M2 phenotype) for 48 h in the presence of BB-Cl. **C** Enriched functional network analysis of citrullinated sites in THP-1 macrophages, as generated by PINE. **D** Normalized citrullinated peptide quantities of NNTQVLINCR47NNK (SNRPD2) and **E** CDSSPDSAEDVR143K (AHSG) in THP-1 macrophages treated with siPAD2 or scrambled siRNA control (siCON) and LPS (M1 phenotype). *p < 0.05; *n* = 5 or 6

To confirm whether PAD activation was necessary and sufficient for THP-1 polarization, we next identified the citrullinated targets of PAD. Using mass spectrometry analysis based on a hypercitrullinated library, we identified 192 citrullinated sites, 180 citrullinated peptides and 152 citrullinated proteins in THP-1 macrophages (Supplemental Table 3A). We identified a modest 21 citrullinated peptides and proteins in BB-Cl-treated THP-1 macrophages polarized to the M1 and M2 phenotypes (Supplemental Table 3A), whereas in PAD2-silenced macrophages, we identified 66 citrullinated peptides and 61 citrullinated proteins (Supplemental Table 3B). The identified and quantified citrullinated proteins in THP-1 macrophages were engaged in signalling by interleukins, neutrophil degranulation, programmed cell death, DNA and RNA processing, proteasomal degradation, platelet activation and VEGF signalling, among others (Fig. 7C). The expression levels of some citrullinated peptides were significantly different between biological conditions, including X-ray repair cross-complementing protein 6 (XRCC6 or Ku70), plectin, small nuclear ribonucleoprotein Sm D2 (SNRPD2) and alpha-2-HS-glycoprotein (AHSG or fetuin-A)(Fig. 7A, B, D and E, respectively). The citrullination of Ku70 on arginine 115 (R115) was downregulated, whereas the citrullination of plectin on arginine 3039 (R3039) was upregulated in LPS-treated proinflammatory THP-1 macrophages (Fig. 7A and B). Furthermore, the citrullination of SNRPD2 on arginine 47 (R47) was decreased, and the citrullination of fetuin-A on arginine 143 (R143) was increased in PAD2-silenced macrophages (Fig. 7D and E).

## Discussion

PADs are a group of hydrolase enzymes expressed in immune cells, and PAD4 and PAD2 are primarily expressed at high levels in neutrophils, monocytes, and macrophages [5, 6]. Although both isoforms are involved in the regulation of inflammatory processes within immune cells, including ETosis in neutrophils [7] and macrophages [8] and pyroptosis mediated by NLRP3 inflammasome activation [9], the detailed mechanism has not been investigated. To address this gap, the THP-1 monocytic cell line was differentiated into macrophages in vitro and then further activated to the M1 or M2 phenotype. The mRNA expression of PAD1, PAD2 and PAD4 was detected in THP-1 macrophages, and PAD2 was the most abundant isoform. Importantly, PAD2 was highly upregulated in M1 macrophages but strongly downregulated in M2 macrophages. Notably, PAD1 and PAD4 were also upregulated in proinflammatory M1 macrophages. Furthermore, for the first time, we reported that PAD modulates the polarization of THP-1 macrophages to proinflammatory M1 and anti-inflammatory M2 phenotypes.

Our results further showed that PAD inhibition decreased proinflammatory M1 markers (TNF-α and IL-6) and increased anti-inflammatory M2 markers (MRC1 and ALOX15) in THP-1 macrophages stimulated with LPS or IL-4. We did not observe decreased levels of IL-1β in the supernatant of LPS-stimulated THP-1 macrophages, which suggests that in our experiments, BB-Cl-amidine did not block NLRP3 inflammasome activation. This finding is contrary to Mishra et al., which showed the blockage of NLRP3 inflammasome activation and IL-1β release after inhibiting PAD activity with Cl-amidine in bone marrow-derived macrophages (BMDMs) [9]. We hypothesize that these differences could be explained by the use of PAD inhibitors that are stronger and less susceptible to proteolysis, a different cell line or a different method of macrophage differentiation. Herein, we reported that inhibiting PAD expression in proinflammatory THP-1 macrophages was associated with the downregulation of several proteins involved in the activation of NF-κβ, such as IKBKG, PRKCB, IKK2, UBE2V1, IFI35, MAVS, EIF2AK2 and RIPK1 (Fig. 4A) [19–26], as determined by DIA-MS proteomic methods. NF-κβ is a rapid-acting transcription factor that plays an important role in regulating the immune response to infection and the production of proinflammatory cytokines [27]. NF-κβ is constantly active in many chronic inflammatory diseases [28]. Moreover, RIPK1 is a key regulator of necroptosis and promotes the production of the proinflammatory cytokines IL-6 and TNF-α [29]. Importantly, BB-Cl-amidine administration decreased the expression of IL-6 and TNF-α at both the mRNA and protein levels in proinflammatory M1 macrophages, which may be associated with the downregulation of RIPK expression. Furthermore, we observed decreased expression of STING1 in both M1 and M2 macrophages after treatment with the PAD inhibitor BB-Cl-amidine. STING promotes the production of type I interferon and activates several transcription factors, such as NF-κβ [30]. STING also contributes to the proinflammatory M1-like macrophage profile by stabilizing HIF-1α, which enhances glycolysis, NOS production and inflammasome activation [31].

Notably, inhibiting PAD with BB-Cl-amidine increased the M2 polarization of macrophages. This effect was related to the downregulation of TXNIP and caspase-1 expression. TXNIP activates the NLRP3 inflammasome and can facilitate M1 polarization but might inhibit M2 polarization [32]. In turn, caspase-1 is responsible for IL-1β and IL-18 production as well as pyroptosis induction [33]. Decreased protein expression of TXNIP and caspase-1 in BB-Cl-amidine-treated, IL-4-stimulated macrophages favours the activation of THP-1 macrophages towards an anti-inflammatory phenotype. Based on these findings, we hypothesize that the inhibition of PAD activity alters the M1 phenotype by downregulating NF-κβ pathway and favours M2 polarization. It is tempting to hypothesize that BB-Cl-amidine is a potential therapeutic agent for treating chronic inflammatory diseases.

To test our hypothesis, PAD2 in THP-1 macrophages was silenced by siRNA. Silencing PAD2 upregulated the mRNA expression of anti-inflammatory markers characteristic of the M2 phenotype (MRC1, FCER2, ALOX15) in both control cells and IL-4-stimulated THP-1 macrophages. Interestingly, silencing PAD2 did not change the mRNA expression of the proinflammatory markers IL-1β and TNF-α, except for IL-6 expression. In our study, the level of IL-6 in the supernatant of proinflammatory M1 macrophages increased. This might be a result of NF-κB activation, as it has been previously shown that PAD2 suppresses NF-κB activity by interacting with IKKγ, an essential regulatory subunit of the IκB kinase complex [34]. Our results contradict those of Yu et al., who demonstrated the promotion of IL-1β, IL-6 and TNF-α production in U937 macrophages with *Pad2* knockout [6]; however, the cell types, cell origin and methodology cannot be directly compared, which suggests dynamic regulation of PAD isoforms, and potentially citrullination.

Furthermore, our data suggest that PAD2 regulates THP-1 macrophage polarization to the anti-inflammatory M2 phenotype, which may explain the low expression levels of PAD2 in M2 macrophages. Notably, silencing PAD2 in THP-1 macrophages upregulated pathways related to the antiviral innate immune response and interferon signalling. MX1, MX2, OAS1, OAS2, OAS3, IFITM3, ISG15, BST2 and RIG-I levels were increased in PAD2-silenced cells. Interestingly, RIG-I, which is a sensor of cytoplasmic viral nucleic acids and leads to the generation of type I interferons when activated, was also found to be involved in IL-6 production [35]. Moreover, silencing PAD2 augmented the level of IL-6 in LPS-treated THP-1 macrophages. BB-Cl-amidine treatment of THP-1 macrophages decreased proteins involved in interferon signalling, which is consistent with other studies showing a decrease in the expression of type I interferon-regulated genes in response to BB-Cl-amidine administration in a systemic lupus erythematosus mouse model [5]. Thus, further investigations are needed to elucidate the role of PAD2 and other PAD isoforms in antiviral innate immune responses and interferon signalling.

Herein, we demonstrated that PAD2 was involved in the regulation of anti-inflammatory M2 macrophages, which was associated with the upregulation of CD74 expression. CD74 is a cell surface receptor for the immunomodulatory cytokine macrophage migration inhibitory factor (MIF), which plays a role in the regulation of macrophage function. It has been shown that MIF–CD74 activation is important for IL-4-induced M2 macrophage polarization in malignant diseases and parasitic infection through the TLR4-PI3K-Akt pathway [36–38]. However, other studies have shown contradictory effects of MIF on the induction of M1 macrophage polarization [39]. In our study, silencing PAD2 or pharmacological inhibition of PAD isoforms in M1 macrophages led to the upregulation of the MIF receptor CD74. In contrast, LPS stimulation caused the downregulation of CD74 expression in THP-1 proinflammatory M1 macrophages (Supplemental Tables 1 and 2). Whether the increase in CD74 protein expression is related to PAD2 inhibition and involved in the polarization of macrophages to the M2 phenotype requires further investigation.

Importantly, we first identified 192 citrullinated sites on 180 citrullinated peptides corresponding to 152 citrullinated proteins in M1 and M2 THP-1 macrophages using a hypercitrullinated library approach combined with DIA-based LC-MS/MS. The identified and quantified citrullinated proteins are involved in signalling by interleukins, neutrophil degranulation, programmed cell death, DNA and RNA processing, proteasomal degradation, platelet activation and VEGF signalling (Fig. 7). The expression levels of some citrullinated proteins, including Ku70 (XRCC6), plectin (PLEC), small nuclear ribonucleoprotein Sm D2 (SNRPD2), and fetuin-A (AHSG), were different between biological conditions. Ku70 is a single-stranded DNA-dependent ATP-dependent helicase that participates in double-strand break repair, chromosome translocation and activation of the innate immune system [40]. Interestingly, it has been observed that Ku70 and Ku80 promote LPS-induced NF-κB activation and proinflammatory cytokine production in human macrophages and monocytes [41]. In our study, citrullinated Ku70 (R115) levels were downregulated in LPS-stimulated THP-1 macrophages. In turn, citrullinated plectin (R3039) was incresed in LPS-treated proinflammatory macrophages. Plectin is an important component of the cytoskeleton; it responds to mechanical forces and interlinks intermediate filaments with microtubules [42]. Notably, citrullination of cytoskeletal proteins such as vimentin, fibrin, collagen II and filaggrin has been reported in various autoimmune diseases [3]. It has been shown that citrullination of cytoskeletal proteins has functional consequences on cell physiology; for example, the citrullination of collagen II affects integrin-mediated cell adhesion [43], and the citrullination of vimentin mediates the development and progression of lung fibrosis [44].

Our results also indicated the increased citrullination of fetuin-A (R143) in PAD2-silenced macrophages. Fetuin-A is a circulating glycoprotein produced by liver and adipose tissue. It acts as a chemoattractant for macrophage infiltration into adipose tissue and the conversion of macrophages to the M1 phenotype [45]. Recent evidence indicates that fetuin-A is a crucial factor that modulates tissue inflammation and fibrosis, as well as a systemic indicator of acute inflammatory disease [46]. Interestingly, several studies have demonstrated an association between fetuin-A serum levels and rheumatoid arthritis disease activity [47]; however, a direct link between the citrullinated form of fetuin A and rheumatoid arthritis has never been presented. The small nuclear ribonucleoprotein Sm D2 belongs to the small nuclear ribonucleoprotein core protein family. It plays pivotal roles in pre-mRNA splicing as a core component of spliceosomal U1, U2, U4 and U5 small nuclear ribonucleoproteins (snRNPs), which are the building blocks of the spliceosome [48]. Importantly, proteins of the U1 small nuclear ribonucleoprotein particle (U1 snRNP) are among the most immunogenic molecules in patients with systemic lupus erythematosus and mixed connective tissue disease [49, 50]. A citrullinated peptide panel has been described for the diagnosis of systemic lupus erythematosus using protein microarrays [51].

Taken together, our results highlight PAD2 as a key regulator of inflammation by modulating macrophage polarization. However, the functional roles of the identified novel citrullinated sites in THP-1 macrophages and their involvement in the regulation of the immune response require further study.

Our study has several limitations. We investigated the influence of a pan-PAD inhibitor and PAD2 silencing on THP-1 macrophage polarization. However, we did not perform experiments in which both PAD1 and PAD4 were silenced in parallel with PAD2 to account for the compensatory effects of other PAD in the absence of PAD2.

## Conclusions

In summary, our study showed that the expression levels of PAD2, PAD1 and PAD4 were increased in THP-1 macrophages activated to the proinflammatory M1 phenotype and decreased in anti-inflammatory M2 macrophages. We showed that the pan-PAD inhibitor BB-Cl-amidine decreased the polarization of THP-1 macrophages to the M1 phenotype and increased activation to the M2 phenotype. This effect was mediated by the downregulation of the NF-κβ pathway. Our results further showed that silencing PAD increased the polarization of THP-1 macrophages to the M2 phenotype and upregulated proteins related to the antiviral innate immune response and interferon signalling. These results suggested that specifically inhibiting PAD2 could be a novel therapeutic strategy. Importantly, we identified 192 citrullination sites in THP-1 macrophages, and the majority of the citrullinated proteins belong to signalling pathways mediating inflammation, cell death and DNA/RNA processing. However, further investigations are needed to elucidate the exact role of PAD and citrullination in proinflammatory and anti-inflammatory macrophages.

## Methods

### THP-1 cell culture

The human THP-1 monocytic cell line was obtained from ATCC (Manassas, VA, USA) and grown in a humidified incubator containing 5% CO_2_ and 95% air at 37 °C in RPMI 1640 medium (Gibco, Waltham, MA, USA) supplemented with 10% foetal bovine serum (FBS, Gibco, Waltham, MA, USA) and streptomycin (100 μg/ml)/penicillin (100 U/ml). To differentiate THP-1 monocytes into macrophages the cells were placed in 6-well plates (1.5 × 10^6^ cells per well) in 3 ml of culture medium and treated with 100 nM phorbol 12-myristate 13-acetate (PMA, Sigma Aldrich, St. Louis, MO, USA) for 48 hrs [52]. After 3 days of rest THP-1 macrophages were polarized for 48 h with 10 ng/ml LPS (*Salmonella Minnesota*; InvivoGen, San Diego, CA, USA) or 20 ng/ml IL-4 (R&D Systems, Minneapolis, MN, USA) to M1 and M2 macrophages, respectively [53]. The control wells were subjected to the same environmental conditions as the stimulated wells. The pan-PAD inhibitor – BB-Cl-amidine (100 nM) was added 30 min before stimulation with LPS or IL-4. The toxicity of BB-Cl-amidine was evaluated by MTT assays at 48 h.

### MTT metabolism assay

To assess THP-1-cell viability after 48 h of BB-Cl-amidine treatment, cells were seeded in a 96-well plate at a density of 50,000 cells per well. Then, 10 μL of a 10 mg/ml MTT solution was added to each well and incubated at 37 °C for 1 hour. Next, the medium was aspirated, 200 uL of DMSO was added, and the plate was shaken for 1 h to dissolve the dye. The absorbance was measured at 570 nm using a Synergy™ 2 microplate reader (BioTek Instruments Inc., Winooski, VT, USA).

### Silencing PAD2

To knockdown PAD2 in THP-1 macrophages, Lipofectamine® RNAiMAX reagent and 25 pmol Silencer®Select PADI2 siRNA (assay ID: s223214)(Thermo Fisher Scientific, Waltham, MA, USA) were used according to the manufacturer’s instructions. Silencer®Select Negative Control #2 (assay ID: 4390846) (Thermo Fisher Scientific, Waltham, MA, USA) was used as the scrambled siRNA negative control. Transfection was performed on Day 2 in resting macrophages for 24 h in RPMI 1640 medium without FBS and antibiotics in 6-well plates.

After stimulation, the cell supernatant was collected, passed through 0.22 μm filters and frozen. THP-1 macrophages were lysed in lysis buffer for further experiments.

### Quantitative reverse transcription polymerase chain reaction

Activation of THP-1 macrophages to the M1 and M2 phenotypes was determined by real-time PCR. The expression levels of proinflammatory genes (IL-1β, IL-6, TNF-α), anti-inflammatory genes (mannose receptor C-type 1 (MRC1), Fc epsilon receptor II (FCER2), arachidonate 15-lipoxygenase (ALOX15)) and PAD isoforms (PAD1, PAD2, PAD3, PAD4) in THP-1 macrophages were determined according to previously described protocol [54]. Briefly, RNA was isolated using the ReliaPrep™ RNA Cell Miniprep System (Promega, Madison, WI, USA) and transcribed to cDNA with the High Capacity cDNA Reverse Transcription Kit (Thermo Scientific, Waltham, MA, USA). Commercially available primers from Bio-Rad (Hercules, CA, USA) (apart from primers for PAD3: 5’CTGGATTGCGACCTGAACTG3’ (forward): 5′ TGTGGTCATCAAAGAGGGCT 3′ (reverse) and PAD4: 5′ ACTCTCCAAGGAACAGAGG 3′ (forward), 5′ GGTATTCCTTGCCCCTGACT 3′ (reverse)) and 2x SsoAdvanced™ Universal SYBR® Green Supermix (Bio-Rad, Hercules, CA, USA) were used for real-time PCR. Analysis of relative gene expression was performed by the CFX96 Touch Real-Time PCR Detection System (Bio-Rad, Hercules, CA, USA) with GAPDH as an internal reference gene, and the data were analysed using the 2^–∆∆Ct^ method in an Excel spreadsheet.

### Enzyme-linked immunosorbent assay (ELISA)

The concentrations of proinflammatory cytokines (IL-1β, IL-6, TNF-α) in the supernatant of THP-1 macrophages were measured by a RayBio® Human IL-1 beta ELISA Kit, RayBio® Human IL-6 ELISA Kit and RayBio® Human TNF-alpha ELISA Kit (RayBiotech, Norcross, GA, USA) according to the manufacturer’s instructions.

### Seahorse real-time cell metabolic analysis

Mitochondrial respiration in THP-1 macrophages activated to the M1 and M2 phenotypes in the presence of BB-Cl-amidine was measured as the oxygen consumption rate (OCR) using a Seahorse XF96 Metabolic Flux Analyser (Agilent Technologies, Santa Clara, CA, USA). THP-1 monocytes were differentiated into macrophages in Seahorse XF96 cell culture plates at a density of 40,000 cells per well. THP-1 macrophages were stimulated with 10 ng/ml LPS or 20 ng/ml IL-4 in the presence of 100 nM BB-Cl-amidine for 24 h. The Seahorse XF Cell Mito Stress Test was performed according to the manufacturer’s protocols. The medium was replaced with XF Base Medium (Agilent, Santa Clara, CA, USA) supplemented with 25 mM glucose, 1 mM sodium pyruvate, and 2 mM L-glutamine (pH 7.4) followed by incubation at 37 °C in a non-CO_2_ incubator for 1 hour. Oligomycin (1 μM), carbonyl cyanide phospho-(p)- trifluoromethoxy phenylhydrazone (FCCP) (1 μM), and rotenone/antimycin A (0.5 μM) were subsequently injected into the wells. At the end of the analysis, the medium was removed, and the cells were lysed in RIPA buffer. The protein concentration was measured by the Pierce™ BCA Protein Assay Kit (Thermo Scientific, Waltham, MA, USA) and used to normalize the OCR. The data were analysed with Wave (version 2.6.1) software (Agilent Technologies, Santa Clara, CA, USA).

### Liquid chromatography–tandem MS (LC-MS/MS) analysis of THP-1 macrophages

THP-1 macrophages were lysed in a buffer containing 0.1 M Tris-HCl, pH 7.6, 2% sodium dodecyl sulfate, and 50 mM dithiothreitol (Sigma Aldrich, St. Louis, MO, USA) at 96 °C for 10 min. The protein concentration was determined by a Pierce 660 nm Protein Assay Kit (Thermo Scientific, Waltham, MA, USA). Seventy micrograms of protein was digested overnight using the filter-aided sample preparation (FASP) method [55, 56] with endoproteinase Lys-C (enzyme-to-protein ratio 1:35) as a digestion enzyme. Next, the samples were purified with C18 Ultra-Micro SpinColumns (Harvard Apparatus, Holliston, MA, USA). For macrophage spectral library preparation equal amounts of peptides from all samples were subjected to a high-pH fractionation protocol on C18 Micro SpinColumns (Harvard Apparatus, Holliston, MA). Fractionation was carried out in 50 mM ammonium formate buffer (pH 10) with 12 consecutive elution steps with 5, 10, 12.5, 15, 17.5, 20, 22.5, 25, 27.5, 30, 35 and 50% acetonitrile in 50 mM ammonium formate buffer (pH 10). All samples and library fractions were dissolved in 0.1% formic acid and 5% acetonitrile at a concentration of 0.5 μg/μl and spiked with the iRT peptides (Biognosys, Schlieren, Switzerland).

One microgram of peptide was injected into a PepMap100 RP C18 75 μm i.d. × 25 cm column (Thermo Scientific, Waltham, MA, USA) via a PepMap100 RP C18 75 μm i.d. × 2 cm trap column (Thermo Scientific, Waltham, MA, USA) and separated using a 1 to 40% B phase linear gradient (A phase - 2% ACN and 0.1% FA; B phase - 80% ACN and 0.1% FA) with a flow rate of 300 nL/min on an UltiMate 3000 HPLC system (Thermo Scientific, Waltham, MA, USA) coupled to a TripleTOF 6600+ (Sciex, Framingham, MA, USA) mass spectrometer. The nanoelectrospray ion source (Optiflow, Sciex, Framingham, MA, USA) parameters were as follows: ion spray voltage: 3.2 kV; interface heater temperature (IHT): 200 °C; ion source gas 1 (GS1): 10; and curtain gas (CUR): 25. For DDA acquisition, spectra were collected for 135 min in full scan mode (350–1400 Da), followed by one hundred CID MS/MS scans of one hundred of the most intense precursor ions from the preceding survey full scan exceeding 100 cps intensity under dynamic exclusion criteria. For DIA acquisition, spectra were collected for 100 min in full scan mode (400–1250 Da), followed by one hundred SWATH MS/MS scans using a variable precursor isolation window approach, with m/z windows ranging from 6 to 90 Da.

DDA-MS data were searched against the human UniProt database and MaxQuant Contaminants list using the Pulsar search engine in Spectronaut software (Biognosys, Schlieren, Switzerland) [57] with the following parameters: ± 40 ppm mass tolerance on MS1 and MS2 levels, mutated decoy generation method, Lys-C enzyme specificity, 1% protein and PSM false discovery rate (FDR). The library was generated using 3–6 fragment ions per precursor. The generated human macrophage library was used to analyse DIA-MS data in Spectronaut software. MS data were filtered by 1% FDR at the peptide and protein levels, while quantitation and interference correction were performed at the MS2 level. The data were normalized by a global regression strategy, Q-value percentile data filtering was set at 50%, and global imputation for missing values was performed. Statistical analysis of differential protein abundance was performed at both the MS1 and MS2 levels [58] using unpaired t-tests with multiple testing correction after Storey [59].

### Constructing protein-protein interaction (PPI) networks

Functional groupings and pathway analysis were performed using PINE (Protein Interaction Network Extractor) software [60] with the STRING and GeneMANIA databases, a score confidence (0.4) and ClueGO *p* value cut-off < 0.05. The mass spectrometry data have been deposited to the ProteomeXchange Consortium via the PRIDE partner repository [61] with the dataset identifier PXD034591.

### Mapping citrullination sites using a hypercitrullinated library approach

To prepare a hypercitrullinated library, THP-1 macrophages were lysed as described previously. Each sample (200 μg) was divided into two tubes (twin samples). To digest proteins and preserve citrullination residues, the FASP method with Lys-C as the digestion enzyme was used. The deimidation reaction was performed on Microcon-30 centrifugal filters during the FASP protocol as described previously [62]. Briefly, one twin sample was treated with the PAD cocktail (cocktail of the five PAD isoforms PAD1, PAD2, PAD3, PAD4, and PAD6; 1:20 ratio, (SignalChem, Richmond, Canada)), while the second sample was treated with H_2_O at the same ratio. All samples were incubated in deimination buffer (100 mM Tris-HCl (pH 8.5), 5 mM CaCl_2_, 0.5 mM DTT) for 2 h at 37 °C. Then, digestion with Lys-C was carried out overnight at 37 °C, and the samples were cleaned on an Oasis HLB plate (Waters, Milford, MA, USA) prior to LC − MS analysis. To fractionate the desalted samples, a Pierce™ High pH Reversed-Phase Peptide Fractionation Kit (Thermo Fisher Scientific, Waltham, MA, USA) was used according to the manufacturer’s instructions with the following modifications: each sample was fractionated into 5 fractions with 7.5, 10, 12.5, 15 and 17.5% ACN in the elution solution. LC-MS analysis was performed as described previously.

A hypercitrullinated library was prepared using SpectraST v.4.0 as described previously [63]. Briefly, all data were searched using X!Tandem Native v.2013.06.15.1, X!Tandem Kscore v.2013.06.15.1, and Comet v.2014.02 rev.2. The search parameters included the following criteria: static modifications of carbamidomethyl (C) and variable modifications of oxidation (M), deamidation (NQ), and citrullination (R). The parent mass tolerance was set at 50 ppm, and the monoisotopic fragment mass tolerance was 100 ppm (which was further filtered to be < 0.05 Da to build the spectral library); LysC peptides with up to two missed cleavages were allowed. The identified peptides were processed and analysed by Trans-Proteomic Pipeline v.4.8 and were validated using PeptideProphet scoring, and the PeptideProphet results were statistically refined using iProphet. All peptides were filtered at an FDR of 1% with a peptide probability cut-off of ≥0.99. To identify citrullinated peptides from DIA runs, a hypercitrullinated spectral library generated by SpectraST was used with CitFinder software to analyse modified-unmodified peptide pairs for physicochemical properties such as ΔRT shift, charge state and neutral loss [63]. Statistical analysis of citrullinated peptides was performed in Perseus [64]. Citrullinated peptide quantities were normalized to their corresponding protein levels. For quantitative analysis, only citrullinated peptides present in at least 50% of each biological group were chosen. Missing values were imputed using a row average imputation method. ANOVA with post- hoc tests and permutation-based FDR correction were used for the statistical analysis of data in Perseus.

### Statistical analysis

Variables are expressed as the mean ± SEM. The equality of variance and normality of the data were checked by the Brown-Forsythe test and Shapiro-Wilk test, respectively. Based on the results, statistical analysis was performed using either t test (two groups), ordinary one-way ANOVA, Brown-Forsythe and Welch ANOVA or Kruskal-Wallis tests with correction for multiple comparisons by controlling the False Discovery Rate (two-stage linear step-up procedure of Benjamini, Krieger and Yekutieli) (Graphpad Prism 9.3.1, San Diego, CA, USA). Two-way ANOVA was used for the OCR analysis. Values of *p* < 0.05 (or q-values for proteomics experiments) were considered statistically significant.

## Supplementary Information

**Additional file 1: Supplemental Figure 1.** Quality control of MS runs of THP-1 macrophages activated to M1 and M2 phenotype in the presence of pan-PAD inhibitor - BB-Cl. A) Total ion chromatogram (TIC) overlay of all LC-MS runs showed excellent separation reproducibility. B) Protein group identification details across all LC-MS runs. C) Spectral library recovery. D) Coefficient of variations for protein groups across experimental conditions. **Supplemental Figure 2.** Bioinformatic functional network analysis of THP-1 macrophages activated to proinflammatory and anti-inflammatory phenotype. A) Enriched functional network generated by PINE (Protein Interaction Network Extractor) in THP-1 macrophages treated with LPS in comparison to control. B) Enriched functional network generated by PINE (Protein Interaction Network Extractor) in THP-1 macrophages treated with IL-4 in comparison to control. Activated pathways are shown as orange central nodes and inhibited pathways are shown as blue central nodes along with red (upregulated) or blue (downregulated) protein nodes. **Supplemental Figure 3.** Quality control of MS runs of THP-1 macrophages with PAD2 knockout activated to M1 phenotype. A) Total ion chromatogram (TIC) overlay of all LC-MS runs showed excellent separation reproducibility. B) Protein group identification details across all LC-MS runs. C) Spectral library recovery. D) Coefficient of variations for protein groups across experimental conditions.

**Additional file 2: Supplemental Table 1 A.** Differentially expressed proteins in THP-1 macrophages treated with BB-Cl-amidine as compared to control group (q < 0.05, *n* = 5). **Supplemental Table 1 B.** Differentially expressed proteins in THP-1 macrophages treated with LPS as compared to control group (q < 0.05, *n* = 5). **Supplemental Table 1 C.** Differentially expressed proteins in THP-1 macrophages treated with IL-4 as compared to control group (q < 0.05, *n* = 5). **Supplemental Table 1 D.** Differentially expressed proteins in THP-1 macrophages treated with LPS and BB-Cl-amidine as compared to LPS group (q < 0.05, *n* = 5). **Supplemental Table 1 E.** Differentially expressed proteins in THP-1 macrophages treated with IL-4 and BB-Cl-amidine as compared to IL-4 group (q < 0.05, *n* = 5).

**Additional file 3: Supplemental Table 2 A.** Differentially expressed proteins in THP-1 macrophages with silenced PAD2 as compared to scrambled siRNA control group (q < 0.05, *n* = 6). **Supplemental Table 2 B.** Differentially expressed proteins in THP-1 macrophages with silenced PAD2 and treated with LPS as compared to scrambled siRNA control group treated with LPS (q < 0.05, *n* = 6).

**Additional file 4: Supplemental Table 3 A.** All citrullinated peptides quantified in THP-1 macrophages polarized to M1 and M2 phenotype in the presence of BB-Cl-amidine. **Supplemental Table 3 B.** All citrullinated peptides quantified in LPS stimulated THP-1 macrophages with silenced PAD2.

## Data Availability

The proteomic datasets generated during the current study are available in the ProteomeXchange Consortium via the PRIDE partner repository with the dataset identifier PXD034591. The other datasets used and/or analysed during the current study are available from the corresponding author on reasonable request.

## Abbreviations

ACN: Acetonitrile
AHSG: Alpha-2-HS-glycoprotein (fetuin-A)
ALOX15: Arachidonate 15-lipoxygenase
ASC: Apoptosis-associated speck-like protein
BB-Cl: BB-Cl-amidine
BST2: Bone marrow stromal antigen 2
CD74: HLA class II histocompatibility antigen gamma chain
DDA: Data-dependent acquisition
DIA: Data-independent acquisition
EIF2AK2: Double-stranded RNA-activated protein kinase
ETosis: Extracellular trap formation
FA: Formic acid
FASP: Filter-aided sample preparation
FC: Fold change
FCER2: Fc epsilon receptor II
FDR: False discovery rate
IFI35: Interferon-induced 35 kDa protein
IFITM3: Interferon-induced transmembrane protein 3
IFN-γ: Interferon gamma
IKBKG: NF-kappa-B essential modulator
IKK2: Inhibitor of nuclear factor kappa-B kinase subunit beta
IL: Interleukin
ISG15: Ubiquitin-like protein ISG15
LC-MS/MS: Liquid chromatography – tandem mass spectrometry
LPS: Lipopolysaccharide
MAVS: Mitochondrial antiviral-signalling protein
MIF: Macrophage migration inhibitory factor
MRC1: Mannose receptor C-type 1
MS: Mass spectrometry
MX1/2: Interferon-induced GTP-binding protein Mx1/Mx2
NO: Nitric oxide
OAS1/2/3: 2′-5′-oligoadenylate synthase 1/2/3
OCR: Oxygen consumption rate
PAD: Protein arginine deiminase
PINE: Protein interaction network extractor
PMA: Phorbol 12-myristate 13-acetate
PRKCB: Protein kinase C beta type
PTM: Posttranslational modification
RIG-I: Antiviral innate immune response receptor RIG-I
RIPK1: Receptor-interacting serine/threonine-protein kinase 1
siCON: Scrambled siRNA control
siPAD2: Silenced PAD2
SNRPD2: Small nuclear ribonucleoprotein Sm D2
STING1: Stimulator of interferon genes protein
TNF- α: Tumour necrosis factor α
TXNIP: Thioredoxin-interacting protein
UBE2V1: ubiquitin-conjugating enzyme E2 variant 1
XRCC6: X-ray repair cross-complementing protein 6 (Ku70)
