# Supplementary material for: Protein arginine deiminase 2 (PAD2) modulates the polarization of THP-1 macrophages to the anti-inflammatory M2 phenotype
Source: J Inflamm (Lond). 2022 Nov 18;19:20. doi: 10.1186/s12950-022-00317-8 (PMC9675280; doi:10.1186/s12950-022-00317-8)
Supplement: Supplementary file 1 — Additional file 1: Supplemental Figure 1. Quality control of MS runs of THP-1 macrophages activated to M1 and M2 phenotype in the presence of pan-PAD inhibitor - BB-Cl. A) Total ion chromatogram (TIC) overlay of all LC-MS runs showed excellent separation reproducibility. B) Protein group identification details across all LC-MS runs. C) Spectral library recovery. D) Coefficient of variations for protein groups across experimental conditions. Supplemental Figure 2. Bioinformatic functional network analysis of THP-1 macrophages activated to proinflammatory and anti-inflammatory phenotype. A) Enriched functional network generated by PINE (Protein Interaction Network Extractor) in THP-1 macrophages treated with LPS in comparison to control. B) Enriched functional network generated by PINE (Protein Interaction Network Extractor) in THP-1 macrophages treated with IL-4 in comparison to control. Activated pathways are shown as orange central nodes and inhibited pathways are shown as blue central nodes along with red (upregulated) or blue (downregulated) protein nodes. Supplemental Figure 3. Quality control of MS runs of THP-1 macrophages with PAD2 knockout activated to M1 phenotype. A) Total ion chromatogram (TIC) overlay of all LC-MS runs showed excellent separation reproducibility. B) Protein group identification details across all LC-MS runs. C) Spectral library recovery. D) Coefficient of variations for protein groups across experimental conditions. [file 12950_2022_317_MOESM1_ESM.pptx]

## Slide 1
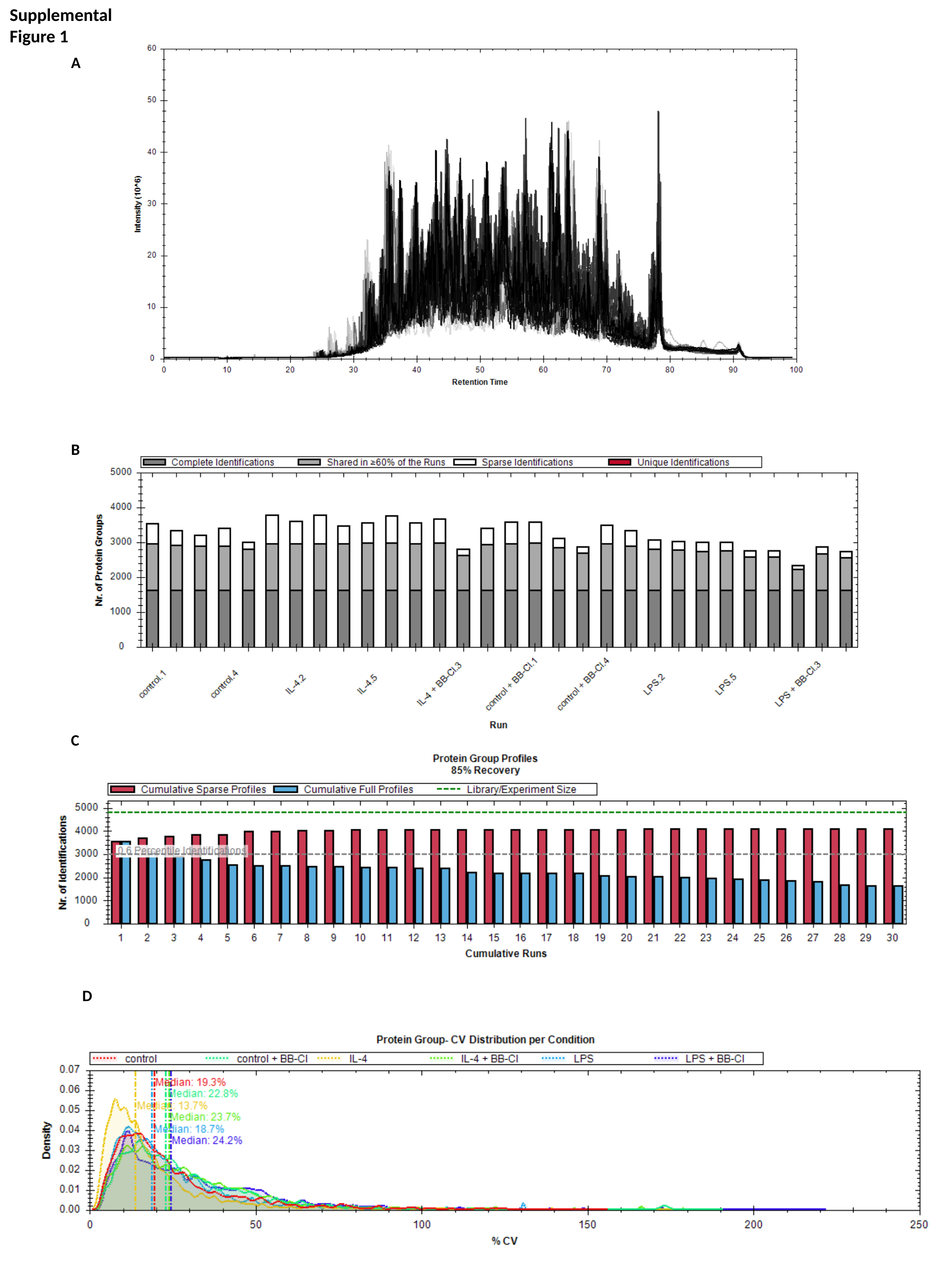

Supplemental Figure 1
A
B
C
D

## Slide 2
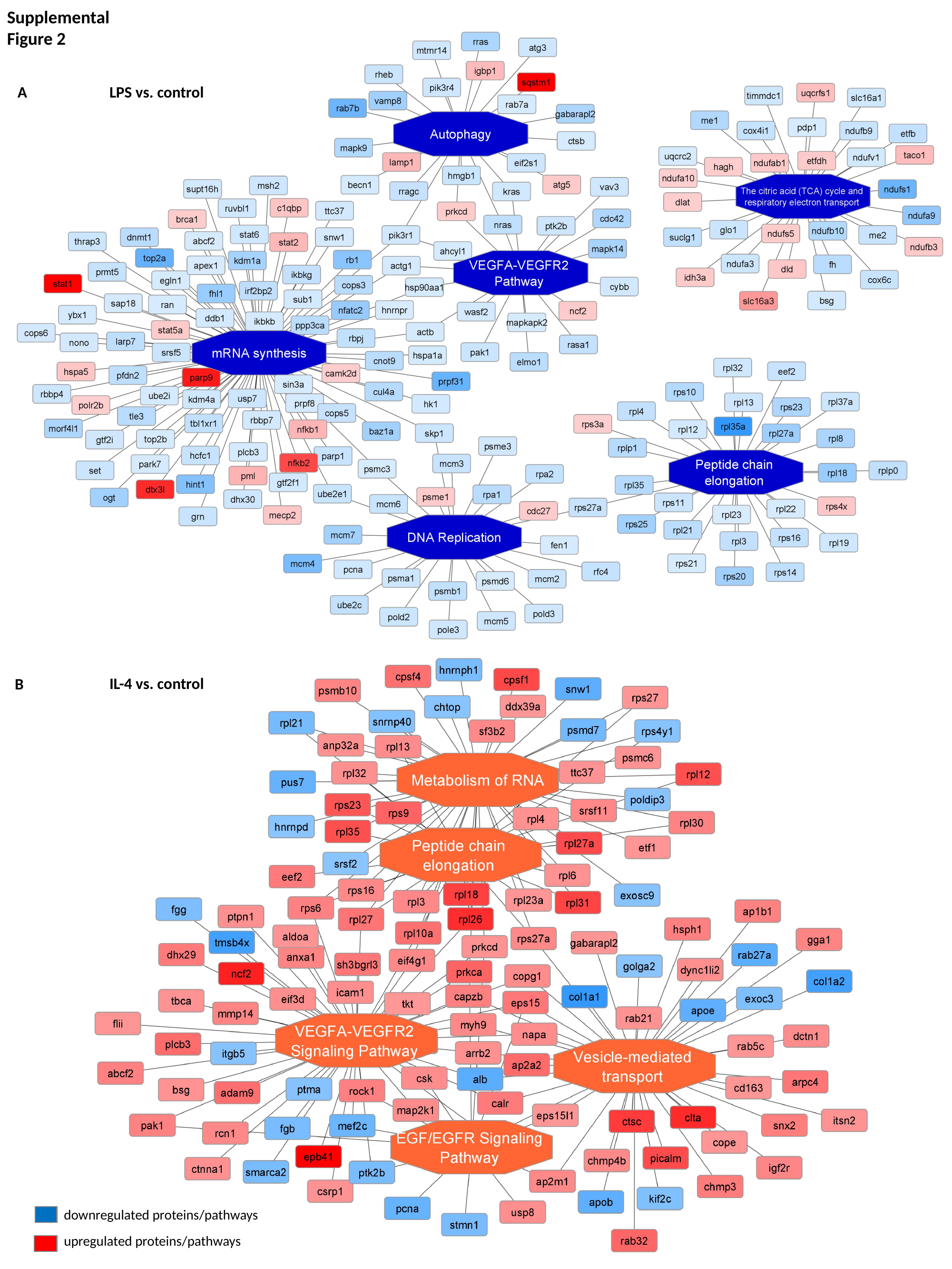

Supplemental Figure 2
A
LPS vs. control
IL-4 vs. control
B
downregulated proteins/pathways
upregulated proteins/pathways

## Slide 3
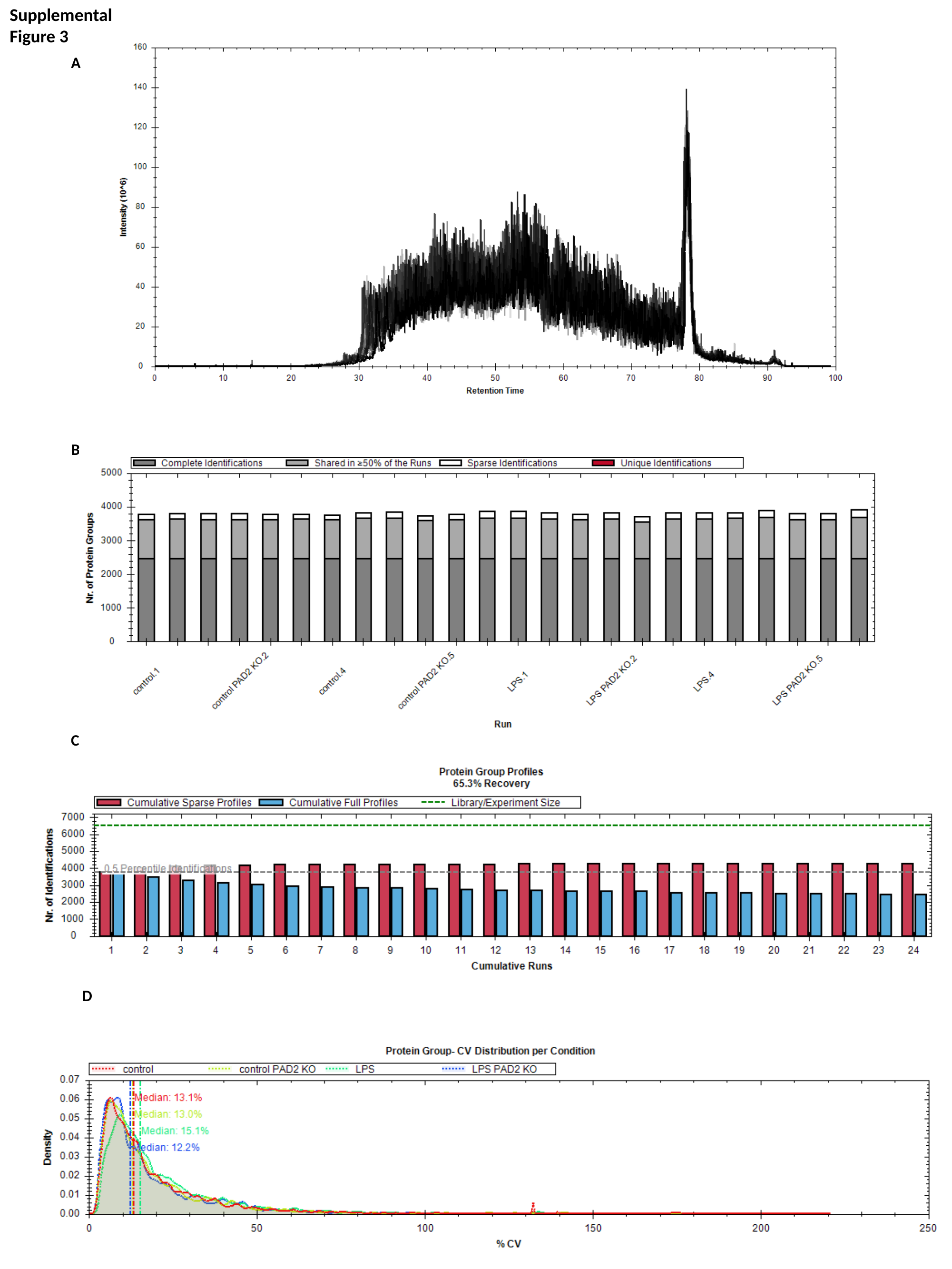

Supplemental Figure 3
A
B
C
D

## Slide 4
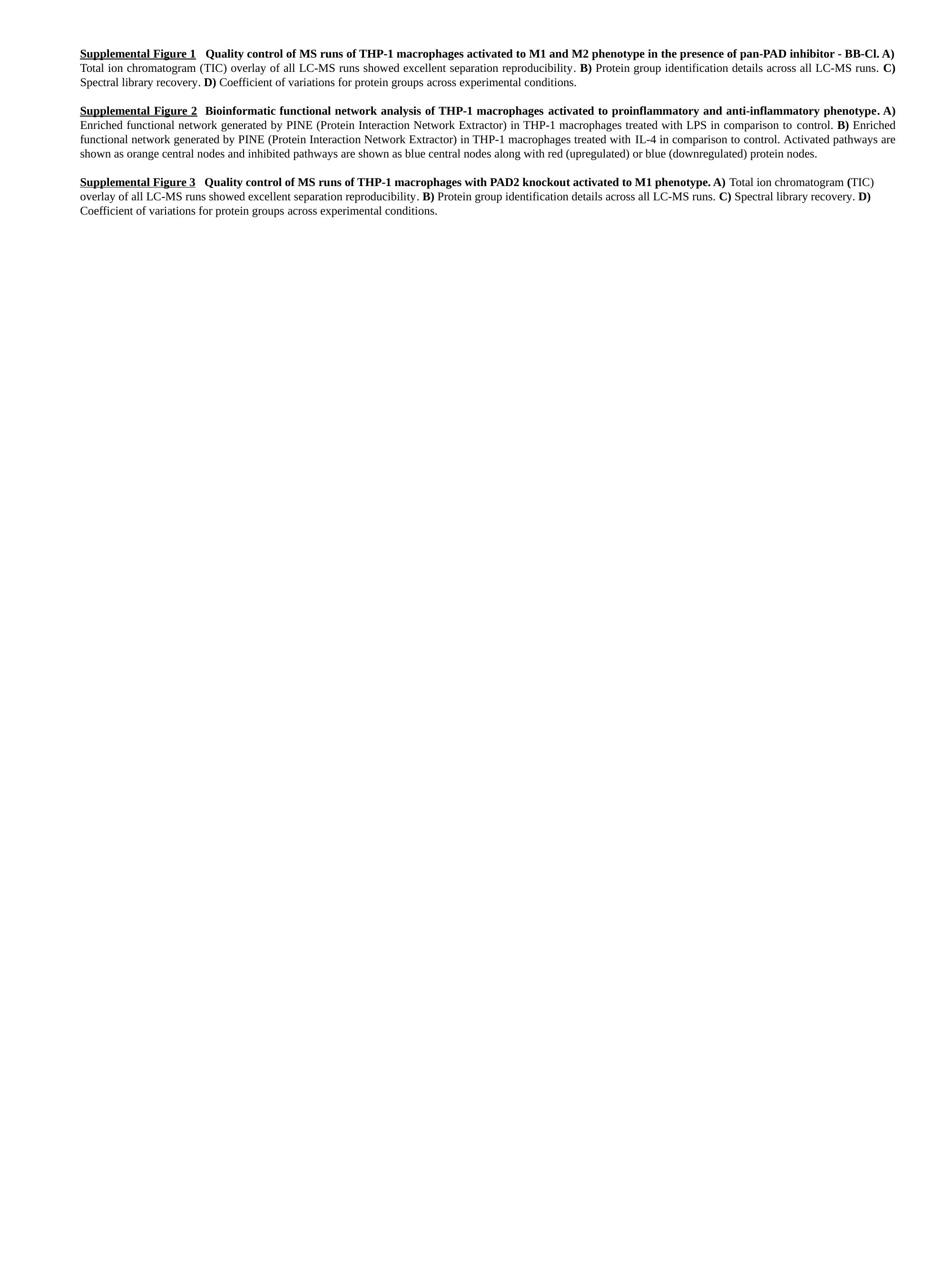

Supplemental Figure 1 Quality control of MS runs of THP-1 macrophages activated to M1 and M2 phenotype in the presence of pan-PAD inhibitor - BB-Cl. A) Total ion chromatogram (TIC) overlay of all LC-MS runs showed excellent separation reproducibility. B) Protein group identification details across all LC-MS runs. C) Spectral library recovery. D) Coefficient of variations for protein groups across experimental conditions.
Supplemental Figure 2 Bioinformatic functional network analysis of THP-1 macrophages activated to proinflammatory and anti-inflammatory phenotype. A) Enriched functional network generated by PINE (Protein Interaction Network Extractor) in THP-1 macrophages treated with LPS in comparison to control. B) Enriched functional network generated by PINE (Protein Interaction Network Extractor) in THP-1 macrophages treated with IL-4 in comparison to control. Activated pathways are shown as orange central nodes and inhibited pathways are shown as blue central nodes along with red (upregulated) or blue (downregulated) protein nodes.
Supplemental Figure 3 Quality control of MS runs of THP-1 macrophages with PAD2 knockout activated to M1 phenotype. A) Total ion chromatogram (TIC) overlay of all LC-MS runs showed excellent separation reproducibility. B) Protein group identification details across all LC-MS runs. C) Spectral library recovery. D) Coefficient of variations for protein groups across experimental conditions.
